# Comparative Proteomic Analysis of Cotton Fiber Development and Protein Extraction Method Comparison in Late Stage Fibers

**DOI:** 10.3390/proteomes4010007

**Published:** 2016-02-03

**Authors:** Hana Mujahid, Ken Pendarvis, Joseph S. Reddy, Babi Ramesh Reddy Nallamilli, K. R. Reddy, Bindu Nanduri, Zhaohua Peng

**Affiliations:** 1 Department of Biochemistry, Molecular Biology, Entomology, and Plant Pathology, Mississippi State University, Mississippi State, MS 39762, USA; hm73@msstate.edu (H.M.); nbrameshreddy@yahoo.co.in (B.R.R.N.); 2 Institute for Genomics, Biocomputing and Biotechnology, Mississippi Agricultural and Forestry Experiment Station, Mississippi State University, Mississippi State, MS 39762, USA; jkpendarvis@email.arizona.edu; 3 College of Veterinary Medicine, Mississippi State University, Mississippi State, MS 39762, USA; Reddy.Joseph@mayo.edu (J.S.R.); bnanduri@cvm.msstate.edu (B.N.); 4 Department of Plant and Soil Sciences, Mississippi State University, Mississippi State, MS 39762, USA; krreddy@pss.msstate.edu

**Keywords:** cotton, fiber elongation, cell wall, comparative proteomics, label-free

## Abstract

The distinct stages of cotton fiber development and maturation serve as a single-celled model for studying the molecular mechanisms of plant cell elongation, cell wall development and cellulose biosynthesis. However, this model system of plant cell development is compromised for proteomic studies due to a lack of an efficient protein extraction method during the later stages of fiber development, because of a recalcitrant cell wall and the presence of abundant phenolic compounds. Here, we compared the quality and quantities of proteins extracted from 25 dpa (days post anthesis) fiber with multiple protein extraction methods and present a comprehensive quantitative proteomic study of fiber development from 10 dpa to 25 dpa. Comparative analysis using a label-free quantification method revealed 287 differentially-expressed proteins in the 10 dpa to 25 dpa fiber developmental period. Proteins involved in cell wall metabolism and regulation, cytoskeleton development and carbohydrate metabolism among other functional categories in four fiber developmental stages were identified. Our studies provide protocols for protein extraction from maturing fiber tissues for mass spectrometry analysis and expand knowledge of the proteomic profile of cotton fiber development.

## 1. Introduction

Being the world’s leading natural textile fiber, the economically-valuable cotton fibers of upland cotton (*Gossypium hirsutum* L.) are unique in the plant kingdom due to their size and chemical composition [1,2]. Each cotton fiber is a single and long (≥2.25 cm) cell originating from the ovule epidermis [2,3,4,5]. Developing near-synchronously during seed development, cotton fiber development consists of four overlapping developmental stages: fiber initiation, cell elongation (primary cell wall synthesis), cell wall thickening (secondary cell wall deposition) and maturation [2,3,5,6]. During primary wall elongation (within 20 days post anthesis (dpa)) and secondary wall deposition and thickening (from 20 to 35 dpa), sizeable amounts of polysaccharide components are synthesized and deposited, producing a cell wall 3 to 4 µm thick, made up of more than 94% cellulose [3,7,8]. These advantageous features make cotton fiber an excellent single-celled model for studying the molecular mechanisms of plant cell elongation, cell wall development and cellulose biosynthesis [2,5,9,10,11]. 

Progress has been made in the large-scale identification of genes and proteins involved in cotton fiber elongation in the last decade [9,12,13]. Several comparative proteome and transcriptome studies during different stages of cotton fiber development have been reported [2,5,8,11,12]. In addition, the transcriptome and proteome comparisons between reduced fiber and fiberless mutants and their respective parental wild-types (WT) have also been conducted [6,14,15,16,17,18,19,20,21,22]. However, due to the recalcitrant nature of cotton fiber, most of the reported studies used young fiber tissue as experimental materials instead of fibers in later developmental stages. Initially, cotton fiber proteins were extracted by directly homogenizing cotton fibers in aqueous buffer followed by organic solvent precipitation [7,23]. However, this method was unsuitable for two-dimensional gel electrophoresis due to the horizontal and vertical streaking and smearing caused by the phenolic and other contaminants co-extracted with proteins [7,23]. In recent years, cotton fiber proteins have been mainly extracted with modifications on the phenol-based procedure [2,5,7,8,10,11,13,20,22,24,25] and by the trichloroacetic acid extraction method, as described by Pang *et al.* [6]. However, there have not been any reports of the successful extraction of cotton fiber proteins for shotgun proteomics from maturing fiber tissues, for example fiber stages after 30 dpa. Despite all of the reported studies, the underlying mechanisms behind fiber initiation, elongation and maturation are still largely unknown [13,21].

The development of novel strategies that optimize protein extraction for cotton fiber cells, particularly the stages after 25 dpa, is critical for using mass spectrometry-based proteomic approaches to study cotton fiber development. Pressure cycling technology (PCT) uses a specifically designed device (Barocycler™) and reaction containers (PULSE™ tubes) to apply cycles of hydrostatic pressure to samples [26,27]. PCT provides a simple, fast, effective and reproducible process to release cellular contents from biological samples [26,27,28]. Previously, it has been shown that the use of PCT increased protein yields from *E. coli*, where PCT extracted 14.2% more total protein than using a standard bead mill [29,30]. Furthermore, 2-DE showed 801 protein spots in the PCT lysate, compared to 760 spots in the bead mill lysate [29]. In mammalian liver tissue, PCT isolated more protein, as well as unique proteins when compared to protein isolation using a Polytron or ground-glass (GG) homogenizers [30,31]. Szabo *et al.* found that PCT-assisted glycan release resulted in the rapid release of asparagine-linked glycans from bovine ribonuclease B, human transferrin and polyclonal human immunoglobulin [32]. It is thought that high pressure alters the protein conformation, pushing water molecules into the protein interior, thus leading to protein unfolding [32,33]. Furthermore, for heat-sensitive molecules, PCT provides an advantage by being able to be conducted at mild temperatures (room temperature to 37 °C) [32,34]. Szabo *et al.* showed that PCT offers several advantages, including not causing decomposition (e.g., desialylation) of the glycan structures, the speed of extraction and the ability to simultaneously process 12 samples at a time [32]. However, the PCT effect on plant protein extraction, particularly the recalcitrant samples, such as cotton fiber, remains to be examined. 

In this report, we identified 1446 proteins in four time points of fiber development (10 dpa, 15 dpa, 25 dpa and 35 dpa). Comparison of the proteomes of different stages of fiber development revealed 287 differentially-regulated proteins, functionally involved in cytoskeleton development, energy/carbohydrate metabolism and cell wall development, among other processes. This study presents the first proteome of the 35 dpa cotton fiber, a highly recalcitrant tissue, and presents a comprehensive proteomic study of fiber development from 10 dpa to 25 dpa. In addition, it provides protocols for protein extraction from maturing fiber tissues for mass spectrometry analysis, and our results considerably expand the knowledge of the cotton fiber proteome during development.

## 2. Experimental Section

### 2.1. Plant Materials

Upland cotton (*Gossypium hirsutum* L.) cultivar Texas Marker (TM)-1 was grown in sunlit, controlled environment chambers, known as soil-plant-atmosphere-research (SPAR) units, located at the Rodney Foil Plant Science Research Center, Mississippi State University, Mississippi, USA, consisting of fine sand as the growing medium [35]. 

### 2.2. Growth Conditions

Detailed operations and controls of SPAR chambers have been previously described [35]. Briefly, the SPAR chamber consisted of a steel soil bin (1 m deep × 2 m long × 0.5 m wide) to accommodate the root system, a Plexiglas chamber (2.5 m tall × 2 m long × 1.5 m wide) to accommodate aerial plant parts and a heating and cooling system connected to air ducts that passed conditioned air to cause leaf flutter through the plant canopy. Variable density shade cloths placed around the edges of the plant canopy, designed to simulate canopy spectral properties, were adjusted regularly to match canopy height and to eliminate the need for border plants. Four rows with five plants per row were maintained in the chamber until harvest. A day/night temperature of 30/22 °C was maintained throughout the experiment. The temperature control was achieved to the desired set points using chilled ethylene glycol supplied to the cooling system via several parallel solenoid valves that were opened and closed depending on the cooling requirements, an electrical resistance heater, which provided short pulses of heat, and a fan, which provided air circulation throughout the chamber [35]. A carbon dioxide concentration of 400 ppm was maintained during the experiment. CO_2_ concentration was monitored and adjusted every 10 s throughout the day and maintained at 400 ± 10 µL·L^−1^ during the daylight hours using a dedicated LI-6250 CO_2_ analyzer (Li-COR, Inc., Lincoln, NE, USA). Plants were well watered with full-strength Hoagland’s nutrient solution [36] three times a day through a programmed drip irrigation system in order to maintain optimum water and nutrient supply throughout the experiment. The SPAR units were supported by an environmental monitoring and control system, which provided automatic acquisition and storage of data, monitored every 10 s throughout the day and night. During the experiment, the incoming daily solar radiation (285 to 2800 nm) outside of the SPAR units was measured with a pyranometer (Model 4–8; The Eppley Laboratory Inc., Newport, RI, USA), which ranged from 1.4 to 27.2 MJ·m^−2^·d^−1^ with an average of 15.6 MJ·m^−2^·d^−1^. The relative humidity (RH) of the chamber was monitored with a humidity sensor (HMV 70Y, Vaisala Inc., San Jose, CA, USA) installed in the returning path of airline ducts. The average relative humidity during the experimental period was 34.5% ± 3.0%. 

Cotton bolls were labeled and tagged on the anthesis day. Harvested fiber time points for protein extraction were 10 days post anthesis (dpa), 15 dpa, 25 dpa and 35 dpa. Fiber samples were collected by hand, immediately frozen in liquid nitrogen and stored at −80 °C until protein extraction was performed. 

### 2.3. Protein Extraction with Phenol

Protein extraction with phenol was performed as previously described [24,37,38,39]. Briefly, 2 grams of cotton fiber were ground under liquid nitrogen using a mortar-pestle; the tissue was suspended in phenol extraction buffer (PEB) containing 0.9 M sucrose, 0.5 M Tris-Cl, 0.05 M EDTA, 0.1 M KCl and 2% 2-mercaptoethanol (added freshly), pH 8.7, and homogenized well. The same volume of phenol was added to the sample, and the sample was homogenized well. The phenol phase was collected by centrifugation at 7000 rpm for 15 min at 4 °C, and phenol extraction was repeated three times. Five volumes of precipitation solution (methanol, 0.1 M ammonium acetate and 1% 2-mercaptoethanol (added freshly)) were added to the final collected phenol phase. To precipitate, the sample was stored at −70 °C overnight. To collect the precipitate, the sample was centrifuged at 12,000 rpm for 15 min at 4 °C. The pellet was collected and washed three times with 70% ethanol. The pellet was vacuum dried and stored at −80 °C. Three replicates were included for extraction methods comparison for the 25 dpa samples and three replicates for comparison were included for differential expression analyses of all of the samples involved.

### 2.4. Protein Extraction with Phenol and PCT for 25 dpa Fiber

Approximately 400 mg of cotton fiber were suspended in PEB buffer with phenol. Tissue was disrupted using the PBI PCT shredder™ following the manufacturer’s instructions, followed by PCT using the Barocycler^®^ NEP2017 pressure cycling instrument (Pressure BioSciences Inc., South Easton, MA, USA) in PCT Shredder PULSE Tubes^TM^ for 60 cycles at 35,000 psi [29,40]. After PCT completion, the remaining phenol extraction steps as described in Section 2.3 were performed. 

### 2.5. Protein Extraction with IEF Reagent for 25 dpa Fiber

Approximately 400 mg of cotton fiber were ground under liquid nitrogen. After tissue disruption, fiber was suspended in isoelectric focusing (IEF) buffer (7 M urea, 2 M thiourea, 4% 3-[(3-cholamidopropyl)dimethylammonio]-1-propanesulfonate (CHAPS)) and vortexed well. After vortexing, samples were centrifuged, and the supernatant was collected. 

### 2.6. Protein Extraction with IEF Reagent and PCT for 25 dpa and 35 dpa Fiber

Approximately 400 mg of cotton fiber were suspended in IEF buffer, followed by PCT, as described in Section 2.4. 

### 2.7. Protein Digestion

Protein concentration was determined using the Bradford assay, and equal amounts of proteins were prepared for digestion. Protein digestion was carried out as previously described [38,39,41,42]. After desalting the digested peptides, the eluted peptides were dried by vacuum and dissolved in 5% acetonitrile (ACN) and 0.1% formic acid. The injection volume for mass analysis was twenty microliters (50 µg digested peptides). 

### 2.8. Shotgun Proteomic Analysis

Chromatography equipment consisted of a Thermo Surveyor HPLC system operated at 500 nL per minute via a split solvent line. A gradient from 5% to 95% acetonitrile in 600 min was used for peptide separation, followed by a 25-min hold at 95% acetonitrile and a 30 min column re-equilibration period; all solvents contained 0.01% formic acid as an ion source. Ten days post anthesis (dpa), 15 dpa and some 25 dpa samples were separated using this gradient for differential expression analysis, while the remaining 25 dpa samples (for method comparison) and 35 dpa samples were analyzed using a 5% to 50% gradient with identical wash and equilibration steps. The latter gradient provided better peptide resolution and was employed in a previous publication [39]. A 0.75 mm by 100 mm BioBasic C18 column (Thermo 72105–100266) was used for peptide separation using both gradients. The Surveyor was coupled with a Thermo LCQ DECA XP Plus mass spectrometer with a stock nanospray ion source. Data were collected over a total duration of 655 min for each sample using MS scans directly followed by three tandem MS/MS scans on the three most intense precursor masses from the full MS scan. Dynamic mass exclusion windows were 2 min long with a repeat count of two. All data collection parameters were identical for both HPLC gradients.

### 2.9. Protein Identification and Statistical Analysis

Spectral files (RAW) were converted to mgf format using msConvert (available in the ProteoWizard tool kit) [43] and searched against the *Gossypium* database downloaded on 22 April 2015 from UniProt, using X!tandem [44]. The database contained 37,423 entries. X!tandem was configured to allow a maximum of 2 missed tryptic cleavage sites and the precursor and fragment mass tolerance were set to 1000 and 500 ppm, respectively. Amino acid modifications included in the database search were single and double oxidation of methionine, carboxymethylation and carbamidomethylation of cysteine, methylation of arginine and lysine residues, phosphorylation of serine and threonine residues, as well as spontaneous water loss after phosphorylation of serine and threonine residues. X!tandem results, by default, consist of individual peptide-spectrum matches, with each match containing information about the corresponding peptide sequence and parent protein. In order to view the results on the protein level, with subsequent peptide sequences listed as a group, they needed to be reorganized. This was accomplished using the Perl programming language to parse the X!tandem results files. As spectrum matches were processed, those with an *E*-value greater than 0.05 were discarded and the remainder organized by corresponding protein. Proteins identified by a single peptide match were also discarded. In order to evaluate the quality of the dataset (false discovery rate), decoy searches were performed using a randomized version of the *Gossypium* protein database and processed using the same Perl logic as before. For all protein search results files, the false discovery rate was less than 0.01 (less than 1%). Results passing all criteria were subsequently organized by dpa and extraction method using Perl. Protein coverage was calculated using Perl. For each protein sequence, an array of zeros the same length as the protein was created. Peptides were string-matched to protein sequence and the coordinates recorded. Elements of the array having the same coordinates were set equal to 1. Once all peptides were processed, the elements were summed, divided by the protein length and multiplied by 100 to calculate coverage. 

### 2.10. Protein Grouping

Since the database used for protein identification contained several species of *Gossypium*, the filtered results included several orthologs identified with the same set of peptides. To reduce redundancy in protein identification, protein grouping was performed using in-house Perl scripts [45] with the following conditions: if two or more proteins were identified with an identical set of spectra in *Gossypium*, only one protein ID (preferentially from *Gossypium hirsutum*) was retained in the protein list, and the remaining protein IDs were grouped together; if two or more proteins were identified with an identical set of spectra in *Gossypium hirsutum*, they were retained in order to account for isoforms in *Gossypium hirsutum*. Only proteins retained after grouping were considered for further analysis. Each group and associated proteins can be found in Supplemental Table S2.

### 2.11. Protein Quantification and Statistical Analysis

To evaluate differences between dpa samples on a proteome level, a differential expression analysis based on peptide elution profiles was performed using Perl. Precursor mass spectra were extracted from the raw data in MS1 format using the msConvert tool from the ProteoWizardtoolkit [43]. Peptide precursor *m*/*z* values were extracted from the previously compiled protein identifications. Elution profiles for peptide-spectrum matches were calculated by parsing each corresponding MS1 file and summing the ion current for that match’s *m*/*z* value within a 1-Da tolerance, effectively integrating the elution profiles. Each trace started at the scan number of the peptide-spectrum match and proceeded both forward and backward until the chromatogram noise level, or a distance of 250 scans, was reached. Multiple peptide-spectrum matches with the same precursor *m*/*z* were only counted once, ensuring the same integral was not counted multiple times. Once all peptide-spectrum matches were processed, intensities were summed for each protein on a per-replicate basis. Proteins not identified in a replicate were represented with the average noise level of the replicate’s chromatogram for further calculations. The reasoning behind this is two-fold: (1) peptides not identified in a replicate could be present at levels at or below the noise level of the chromatogram, causing the mass spectrometer to ignore them; and (2) for calculating expression ratios between lines, zero cannot be in the denominator. Data were normalized using a mode-based technique. First, the mode of the protein intensities for each replicate was calculated, representing the most commonly-occurring protein intensity. Next, for each identified protein, the intensity per replicate was divided by the mode of the same replicate. This ensures that normalization is not affected by the minimum and maximum intensities, which can vary tremendously between replicates. For each protein, the ratio between lines was calculated from the replicate intensities. A Monte Carlo resampling analysis was performed to evaluate the significance of the intensity distribution. For each replicate, a random intensity was generated between and including the minimum noise level and maximum intensity across all replicates. A new ratio between lines was calculated and compared to the original. This process was performed one million times, and the number of times the random ratio was above or below the experimental ratio was recorded. From this, a *p*-value was calculated for each distribution to indicate significance. Expression differences with a *p*-value ≤ 0.05 were considered significantly differentially expressed.

## 3. Results

### 3.1. Comparison of Different Protein Extraction Buffers and Tissue Grinding Methods

**Figure 1:**
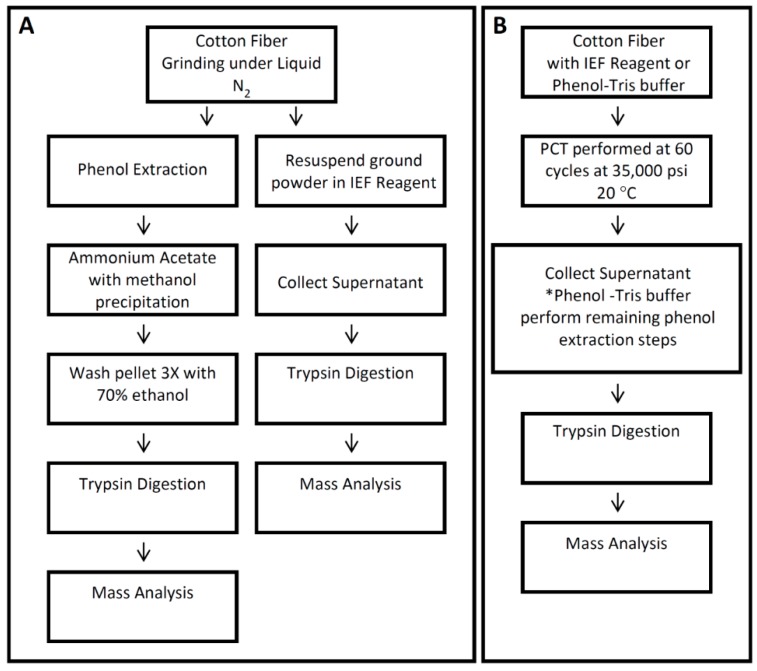
Summary of protein extraction methods used in this study. Cotton fiber proteins from 25 days post anthesis (dpa) fiber were extracted using different protein extraction methods, as shown in (**A**) and (**B**). Cotton fiber proteins were extracted with the mortar-pestle method combined with isoelectric focusing IEF reagent (A), the mortar-pestle method combined with phenol-Tris buffer (A) and with pressure cycling technology (PCT) combined with IEF reagent or phenol-Tris buffer (B). *After PCT combined with phenol-Tris buffer was completed, remaining phenol extraction steps were performed as described in Section 2.3.

Cotton protein extraction for proteomics studies has largely been performed using modifications on the traditional phenol extraction method as reported [2,5,7,8,10,11,13,20,22,24,25] and TCA extraction as described by Pang *et al.* [6]. As cotton fiber tissue matures, however, the protein content decreases, the cell wall thickens and interfering content, such as polysaccharides, polyphenolic compounds, pectin, lipids and waxes, is present, which substantially affect downstream proteomic applications [7,46]. To optimize the protein extraction method for cotton fiber, particularly the fiber from later developmental stages, such as 25 dpa and 35 dpa, we examined different extraction procedures using 25 dpa fibers as shown in Figure 1. 

**Figure 2:**
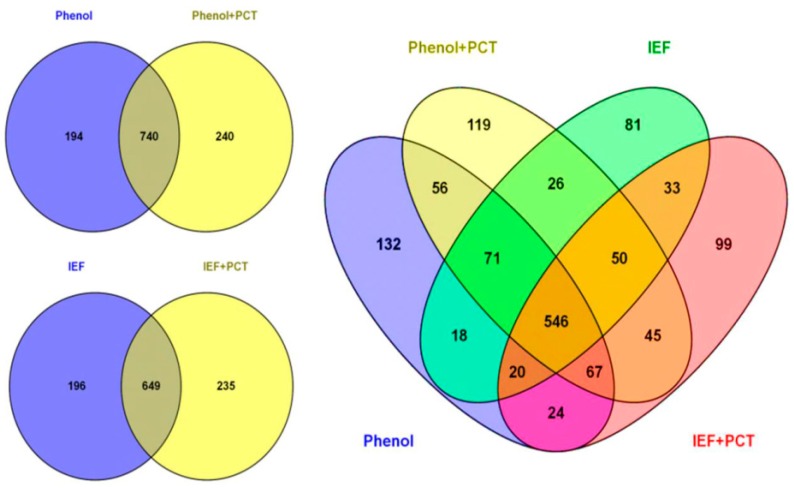
Analysis of 25 dpa cotton fiber proteome obtained using four different extraction methods (phenol, phenol + PCT, IEF and IEF + PCT). Venn diagrams displaying the number of identified cotton proteins in each extraction procedure for 25 dpa cotton fiber and the overlap of identified proteins among extraction procedures. Venn diagrams were generated using the Venny 2.0 tool [47].

We found that pairing the pressure cycling technology with both the IEF buffer and phenol could improve protein identification. Grinding the sample under liquid nitrogen (mortar-pestle extraction) and using the IEF buffer identified 845 proteins. When PCT was used with the IEF buffer, 884 proteins were identified, from which 235 were newly-identified proteins (Figure 2). When phenol extraction was coupled with PCT, 980 proteins were identified, while grinding the sample under liquid nitrogen and using phenol alone identified 934 proteins (Figure 2). 

**Figure 3:**
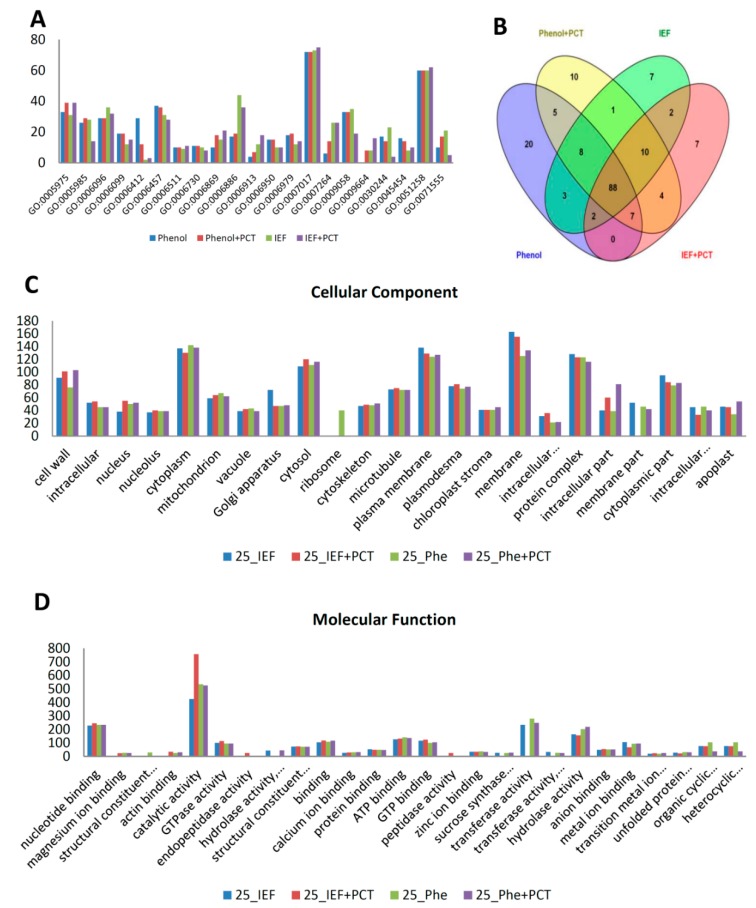
Gene ontology (GO) annotations of proteins identified using four protein extraction procedures in 25 dpa cotton fiber. Protein number, y-axis; gene ontology terms, x-axis. Phe, phenol. Phe+PCT, phenol+PCT. Only predominant GO category terms are shown in figure (**A**). Predominant biological process GO category terms of 25 dpa fiber proteins identified by the four extraction procedures, respectively. (**B**) Venn diagram displaying overlapping biological process category terms across four extraction procedures used for 25 dpa fiber. (**C**) Predominant cellular component category terms for proteins identified in 25 dpa fiber using four extraction methods, respectively. (…) in the x-axis indicates an abbreviated term; complete names of abbreviated cellular component GO terms from left to right include: intracellular membrane-bounded organelle || intracellular organelle part. (**D**) Predominant molecular function category terms for proteins identified in 25 dpa fiber using four extraction methods, respectively. (…) in the x-axis indicates abbreviated terms; complete names of abbreviated molecular function GO terms from left to right include: structural constituent of ribosome || hydrolase activity, hydrolyzing O-glycosyl compounds || structural constituent of cytoskeleton || sucrose synthase activity || transferase activity, transferring glycosyl groups || transition metal ion binding || unfolded protein binding || organic cyclic compound binding || heterocyclic compound binding.

For functional classification of the identified proteins, we carried out gene ontology analysis using the UniProt-GOA database (www.uniprot.org). The proteins were classified by gene ontology annotation based on three categories: biological process, cellular component and molecular function. Analysis of the identified proteins by each of the four extraction methods showed that the majority of the proteins identified in all methods were involved in microtubule-based processes (GO:0007017) and protein polymerization (GO:0051258) (Figure 3A). The majority of the GO terms from these categories overlapped significantly across the four methods, with phenol identifying the most unique biological processes’ GO terms (Figure 3B). Using the GOSlimAuto [48] available at AgBase, the cellular component and molecular function gene ontology terms were compared across the four protein extraction procedures (Figure 3C,D). Although the majority of proteins identified across the four extraction methods did not significantly belong to any one category, a large number did come from the cytoplasm, cytosol, cell wall, plasma membrane, protein complex and membrane (Figure 3C). Interestingly, ribosomal proteins were only identified in the phenol extracted samples (Figure 3C). When looking at the molecular function, the majority of the proteins had catalytic activity and nucleotide binding (Figure 3D). 

### 3.2. Fiber Proteome

We identified 451 proteins with two or more peptides in 10 dpa cotton fiber using the phenol extraction method. Meanwhile, 407 proteins were identified with two or more peptides in the 15 dpa fibers using the same extraction method. For 25 dpa fiber, a total of 1387 proteins were identified with two or more peptides when all four extracting methods were included (Figure 2). In an effort to improve protein identification for later stage fibers, both IEF + PCT and phenol extraction methods were used for 35 dpa fiber. Using these two methods, a total of 785 proteins were identified with two or more peptides. With all protein extraction methods combined in this study, we identified 1446 proteins of the fiber proteome (Supplemental Table S1). 

### 3.3. Functional Analysis of the 35 dpa Proteome

**Figure 4:**
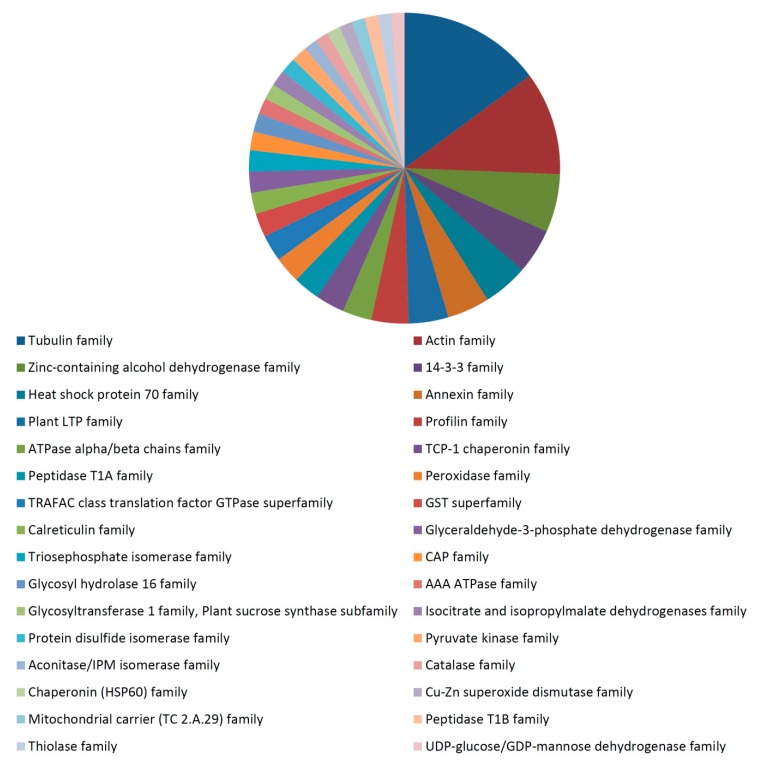
Predominant protein families identified in the 35 dpa cotton fiber proteome. The section size is proportional to the numbers of proteins identified in the category. The name of each category is shown below the chart.

Functional analysis of the total identified proteins in the 35 dpa fiber proteome revealed a strong role of cytoskeletal and energy/carbohydrate metabolism proteins at this fiber stage (Supplementary Figures S1–S3). Major biological processes involving 35 dpa proteins included the metabolic process, oxidation-reduction process, microtubule based process, seed trichome elongation, protein polymerization, response to stress, carbohydrate metabolic process, transport, *etc.* (Supplementary Figure S1). The 35 dpa proteome contained proteins from the cytoplasm, plasma membrane, protein complex, membrane, cytosol, cell wall, plasmodesma, cytoplasmic part, microtubule, mitochondrion, Golgi apparatus, *etc.* (Supplementary Figure S2). The predominant molecular functions of proteins identified in the 35 dpa proteome included catalytic activity, nucleotide binding, transferase activity, hydrolase activity, ATP binding, GTP binding, GTPase activity, structural constituent of cytoskeleton, metal ion binding, *etc.* (Supplementary Figure S3). The proteins in the 35 dpa proteome belonged to a broad range of protein families (Figure 4). The major protein families identified in the 35 dpa proteome included the tubulin family, actin family, zinc containing alcohol dehydrogenase family, 14-3-3 family, heat shock protein 70 family, annexin family, plant LTP family, profilin family, ATPase alpha/beta chains family, *etc.* (Figure 4). Cytoskeletal proteins identified in the 35 dpa proteome included several actin isoforms, as well as alpha and beta-tubulin isoforms and annexin, among others. Energy/carbohydrate metabolism proteins included enolase isoforms, fructokinase (D2D2Z5), glyceraldehyde-3-phosphate dehydrogenase, sucrose synthase 1, 4, 2 and sucrose synthase isoforms B, C and D. The presence of proteins involved in cytoskeletal and carbohydrate metabolism in the 35 dpa proteome is understandable, since cellulose is the major carbohydrate in cotton fiber at maturity [49]. A large amount of energy is needed to produce and deposit the secondary wall cellulose microfibrils [49]. 

The 35 dpa proteome also contained many redox-related proteins. Proteins in this category included 2-nitropropane dioxygenase (D2D302), 3-ketoacyl-CoA thiolase 2 (A0A0B0N1S6), ascorbate peroxidase (C6ZDA9), protein disulfide isomerase (G1ED19), benzoquinone reductase (A3F7Q2), superoxide dismutase (Q3SAW9), catalase isozyme 1, 2 (A0A0B0PAB0, P30567), among others. Several cell wall synthesis-related proteins were also identified, including endo-1,3-beta-glucanase (O23953), several endo-alpha-1,4-glucanase isoforms (F1BWY1, Q4F885, F1BWY2), pectin methylesterase 5 (R9QQU5), UDP glucose pyrophosphorylase (Q6RY01), GRP-like protein 2 (Q0PW29) and fasciclin-like arabinogalactan protein 6, 2, 5, 1 and 4 (A9XTL1, A9XTK7, A9XTL0, B1NHU5, A9XTK9), among others. 

### 3.4. Differentially-Expressed Proteins during Fiber Development

During fiber development, fiber cells undergo a series of morphological and compositional changes. To further examine developmental regulation of the cotton fiber proteome, we examined differentially-expressed proteins in different stages of fiber development. We quantitatively compared the proteome changes in the following stages: 15 dpa *vs.* 10 dpa and 25 dpa *vs.* 15 dpa. A non-labeling quantification method based on peptide spectral intensity was used [50]. Differential expression was only considered for proteins with a *p*-value ≤ 0.05. 

Following the course of fiber development, we found that 287 proteins were differentially expressed in total (Supplemental Table S3). To further analyze the differentially-regulated proteins, functional classification of the differentially-expressed cotton proteins was carried out according to the gene ontology (GO) rules using GOSlimAuto available at AgBase [39,48,51,52]. 

Functional analysis and comparison of the biological processes of the upregulated proteins showed that when 15 dpa *vs.* 10 dpa stages were compared, the predominant biological processes were the oxidation-reduction process, biosynthetic process and sucrose metabolic process, among others (Supplementary Figure S4). The predominant categories of the cellular component of the upregulated proteins in this comparison (15 dpa *vs.* 10 dpa) were cytosol, intracellular and nucleus, among others (Supplementary Figure S5). The predominant molecular function categories of the comparison (15 dpa *vs.* 10 dpa) were oxidoreductase activity, metal ion binding and zinc ion binding, among others (Supplementary Figure S6). When 25 dpa *vs.* 15 dpa stages were compared, the predominant biological processes of the upregulated proteins were the metabolic process, microtubule-based process and protein polymerization, among others (Supplementary Figure S4); the predominant cellular components were microtubule, cytoplasm and cytosol, among others (Supplementary Figure S5); and the predominant molecular function categories were nucleotide binding, GTPase activity and structural constituent of cytoskeleton (Supplementary Figure S6) in the same comparison. 

When 15 dpa *vs.* 10 dpa downregulated proteins were compared, proteins belonging to biological processes, such as metabolic process, transport and plant-type cell wall organization, among others, were the predominant (Supplementary Figure S7). Meanwhile, proteins in membrane, cytoplasm and cell wall were predominant categories of cellular components in the same comparison of the downregulated proteins. (Supplementary Figure S8). Additionally, proteins in catalytic activity, transferase activity and hydrolase activity, among others, were the main molecular function categories in the comparison (15 dpa *vs.* 10 dpa) (Supplementary Figure S9). The 25 dpa *vs.* 15 dpa downregulated protein comparison showed a predominance of proteins involved in the oxidation-reduction process, transport and protein folding, among others (Supplementary Figure S7). The 25 dpa *vs.* 15 dpa downregulated protein comparison showed a predominance of proteins residing in the cytoplasm, plasma membrane and apoplast, among other cellular components (Supplementary Figure S8). The same comparison showed a predominance of proteins involved in transferase activity, nucleotide binding, and catalytic activity, among other molecular functions (Supplementary Figure S9). 

### 3.5. Differential Expression of Cytoskeletal Related Proteins

Identifying proteins related to fiber development and maturation is essential for discovering the cellular network responsible for fiber and cell wall development. In this study, we identified multiple cytoskeletal-related proteins that were differentially regulated, including the actin binding proteins, profilin and actin-depolymerizing factor (ADF). Profilins have been found to be expressed during early cotton fiber development [53,54,55], and when overexpressed in transgenic tobacco cells, they produced an elongated cell with thicker and longer microfilament cables [54,55]. We found that an isoform of profilin (A0A0B0PF49) was downregulated during secondary wall deposition (25 dpa) when compared to an earlier fiber development stage (15 dpa), while actin depolymerizing factor 7 (A1XJ44) was found to be upregulated in 15 dpa when compared to 10 dpa and downregulated in 25 dpa fiber when compared to 15 dpa. Another cytoskeleton binding protein, known as annexin, was found to be downregulated in 15 dpa (Q69DC2, Q8W4Z7, A6MUT1) and 25 dpa fiber (Q69DC2, S5GI78, D2D2Z9, S5G619, M4MX81). Annexins are capable of binding to calcium and lipid membranes, allowing them to play a role in signaling networks, membrane trafficking and cytoskeletal interactions [56,57,58,59,60,61,62,63]. 

We also identified several beta and alpha-tubulin proteins that were differentially expressed, including one that was upregulated in 15 dpa fiber (A0A0B0MX93, tubulin beta-1 chain-like protein). Several others were upregulated in 25 dpa fiber, including alpha-tubulin 3 (Q8H6L9), alpha-tubulin 2 (Q8H6M0), tubulin beta-7 chain (A0A0B0PUW5), tubulin alpha-2 chain (A0A0B0NQH0), beta-tubulin 1 (A6MUS5) and tubulin beta-7 chain (A0A0B0NRH5). Alpha-tubulin 3 (Q8H6L9), alpha-tubulin 2 (Q8H6M0) and tubulin alpha-3/alpha-5 chain-like protein (A0A0B0PKT8) were downregulated in 15 dpa fiber. Other important differentially-regulated cytoskeletal proteins included an actin protein that was downregulated in 15 dpa fiber (A6MUT3); while four other actin proteins showed downregulation in 25 dpa fiber (Q7XZK1, A0A0B0NUJ5, A0A0B0MGZ2 and I1T3U6). 

### 3.6. Differential Expression of Cell Wall-Related Proteins

Many proteins involved in cell wall synthesis and modification were found to be differentially expressed at various time points during the fiber development process. Interestingly, several expansin protein family members showed downregulation during fiber development. For instance, alpha-expansin (B2Z3V3, Q283Q7, Q8LKK3, Q8LKK2), expansin (Q7Y254, O23952), alpha expansin 1 (A6YR48, I1T470, A6YR43, I1T469) and alpha expansin 2 (A6YR66 and A6YR63) were downregulated in 15 dpa fiber.

### 3.7. Differential Expression of Energy/Carbohydrate Metabolism Proteins

Many sucrose synthase isoforms were found to be differentially regulated. For instance, sucrose synthase proteins (I1T4T5, G9BRX6, I1T4T6, H6AC56, H6AC57 and G9BY14) were upregulated in 15 dpa fiber; while in 25 dpa fiber, sucrose synthase isoform C proteins were upregulated (G1JRK6, G1FNX7 and G1FNX4). Some sucrose synthase proteins were also downregulated. For example, sucrose synthase isoform B (G1JRK5), sucrose synthase isoform D (G1JRK7), sucrose synthase Sus1 (I1T4R3), sucrose synthase 2 (G9BRX6) and sucrose synthase (Q9ZRC4, I1T4T5, I1T4T6, H6AC56, H6AC57, G9BY14) were all downregulated in 25 dpa fiber. The large number of differentially-regulated energy/carbohydrate metabolism proteins suggested important roles of these proteins in fiber development, which is consistent with prior reports. Brill *et al.* showed that SusA/B/D isoforms are expressed highly during fiber elongation, falling off during secondary cell wall synthesis [64]. However, SusC is absent at both the transcript and protein levels in early fiber development, but highly expressed in later fiber development [64].

## 4. Discussion

### 4.1. Comparison of Protein Extraction Methods for Recalcitrant Cotton Fiber

Protein extraction from recalcitrant plant tissue is highly challenging due to the abundance of interfering plant chemical compounds and extremely robust cell wall. Efficient and routine study of recalcitrant plant material has been limited due to the high amount of plant secondary metabolites, including phenols, flavonoids, stilbenes, terpenes, tannins and lignins, which negatively affect downstream proteomic efforts [65,66]. Secondary metabolites build up as soluble forms in the vacuoles and are more prevalent in adult mature tissues than in young etiolated tissues [66,67]. For proteome analysis of cotton fiber, an ideal protein extraction method should successfully disrupt the cell wall and reproducibly extract all of the proteins in the proteome, with efficient removal of non-protein contaminants [66]. In the present study, we evaluated plant protein extraction protocols, including phenol extraction and IEF buffer coupled with and without pressure cycling technology (PCT). A comparison of the protein extraction methods was done based on protein identification. 

Considering protein identification, phenol plus PCT identified the largest amount of proteins (980), while phenol extraction came in second, identifying 934 proteins with two or more peptides in 25 dpa fiber. PCT used together with IEF buffer identified 884 proteins; without PCT, 845 proteins were identified in 25 dpa fiber. Our results suggested that to thoroughly study the cotton total proteome, the use of multiple extraction procedures collectively is favorable, because they provide a broader range of coverage of the total proteome. Combining the various protein extraction methods in this study, we identified 1446 proteins (Supplemental Table S1, Supplemental Tables S4 to S7 and Supplemental Figures S10 to S12) and 287 differentially-expressed proteins (Supplemental Table S3) in *G. hirsutum*. Our study provided a comprehensive proteome in *G. hirsutum*, including the proteome of 35 dpa fiber. 

### 4.2. Proteome Studies on 35 dpa Fiber

Although many studies have been performed on different stages of cotton fiber development, the majority of these studies have primarily focused on early fiber stages due to the recalcitrant nature of maturing cotton fiber. Many studies cited the difficulty resulting from the tough secondary cell wall exterior and reduced protein content [7,46]. To the best of our knowledge, this is the first report to present proteome study data on 35 dpa fiber. We identified 785 proteins in the 35 dpa fiber proteome. Seventeen of these proteins were exclusively identified in 35 dpa fiber tissue. These proteins included three uncharacterized proteins (A0A0B0N1M7, A0A0B0MIW5, A0A0B0PNM1), glutamate—glyoxylate aminotransferase 2-like protein (A0A0B0NW95), peroxidase 51-like protein (A0A0B0PPW0), WRKY transcription factor 1-like protein (A0A0B0PEV3), NF-X1-type zinc finger NFXL1-like protein (A0A0B0NHR9), GDSL esterase/lipase CPRD49-like protein (A0A0B0N5L5), quinone oxidoreductase-like protein 2 (A0A0B0P6H8), Biotin carboxyl carrier protein subunit (A8RWF8), alcohol dehydrogenase class-P-like protein (A0A0B0NGV8), 60S acidic ribosomal P1 (A0A0B0PNW5), cell division cycle 48 (A0A0B0NEP6), UDP-glucuronic acid decarboxylase 1 (A0A0B0N8J8), polygalacturonase-inhibiting protein (Q6WMU5), lysosomal beta glucosidase (A0A0B0PUQ5) and beta-D-xylosidase 1-like protein (A0A0B0PLT0). 

### 4.3. Proteome Differential Regulation during Cotton Fiber Development

In this study, we aimed to identify regulatory proteins that were expressed at specific developmental stages. We found 287 proteins that were differentially expressed at one or more of the three time points compared. However, the number of differentially-regulated proteins identified in this study may be inflated due to the presence of homologous proteins in the database, which can share peptides. It is difficult to distinguish whether all of the proteins sharing the same set of peptides are present in the sample or if only some of the proteins are actually present [68]. In future studies, identification of proteotypic peptides for quantitative proteomics would aid in overcoming this setback [68]. Many of the differentially-expressed proteins belonged to functional classes involved in cytoskeletal arrangement, energy/carbohydrate metabolism, stress responses and cell wall. The distribution of the differentially-regulated proteins indicates the cytoskeleton plays a critical role in cotton fiber development; as well as a strong role of energy/carbohydrate metabolism throughout fiber development. This result is plausible, because rapid cell elongation and fiber development require a large amount of energy, as well as cytoskeletal and cell wall dynamic rearrangement [8]. Furthermore, the distribution of the identified proteins shows that cotton fiber cells possess an increased level of redox-related activity at the 15 dpa fiber stage, likely due to H_2_O_2_ content increasing at 15 dpa and peaking at the 20 dpa stage of fiber development [8]. Altogether, protein functional analysis and regulation data indicate that fiber development involves an interplay between cytoskeletal, cell wall and metabolic proteins, creating a complex arrangement of events associated with key fiber developmental stages. Our observations were consistent with prior 2-DE gel-based studies on fiber development and comparison between the fuzzless-lintless mutant and wild-type.

### 4.4. Cytoskeletal Dynamics

Cytoskeletal architecture in cotton fibers plays an essential role in cotton fiber development. Plant cell morphology is largely determined by the highly dynamic actin cytoskeleton [55]. In this study, we found that cytoskeletal proteins were highly differentially regulated during fiber development, which included actin and actin binding proteins profilin and annexin. Actin was found to be downregulated in 15 dpa fiber and 25 dpa fiber when compared to earlier time points. 

The activity of actin is further modulated by actin-modifying proteins, including profilins (PFN), which were found to be upregulated in 15 dpa cotton fiber in this study. Wang *et al.* found that expression of a profilin family member (GhPFN2) was significantly induced during the period of rapid fiber elongation and secondary wall synthesis [55,69]. Another important regulator of actin dynamics, annexin was found to be downregulated in 15 dpa and 25 dpa fiber. In cotton, it was shown that four cotton annexin proteins (AnxGh1: AAR13288, AnxGh2: AAB67993, AnxGhFx:FJ415173, AnxGhF: AAC33305) were present in higher amount in fibers of 10 dpa wild-type plants as compared to the fuzzless-lintless mutant [6,63]. Another actin regulating protein called actin depolymerizing factor (ADF) was found to be upregulated in 15 dpa and downregulated in 25 dpa. Interestingly, it was found that increased expression of profilin 2, as well as suppression of actin depolymerizing factor 1 led to inhibition of fiber elongation and promoted secondary wall formation [69,70,71]. 

Another important family of cytoskeletal proteins known as tubulins, specifically several beta and alpha-tubulin isoforms, were differentially expressed. The bulk of these proteins were upregulated in 25 dpa fiber. During fiber elongation, a minimum of nine β-tubulin genes are preferentially or differentially expressed [72]. A study by Pu *et al.* in cotton fiber suggested that transcription factors, such as *GhMYB109*, control key regulatory processes by modulating microtubules by influencing the expression of *GhTUB1* and *GhACT1* genes, thereby effecting subsequent downstream cellular effects related to the cytoskeleton [72,73]. Therefore, the differential regulation of actin and tubulin variants along with actin-regulating proteins may be part of a complex regulatory network of proteins controlling fiber development. 

### 4.5. Cell Wall Dynamics

Cotton fiber provides a good model for studying the regulation of primary and secondary cell wall development. During rapid fiber elongation, it is known that genes encoding wall-loosening expansin proteins [74,75] are expressed and are associated with quantitative trait loci linked with fiber length [72,76,77]. In this study, expansins were also found to be differentially regulated. The expansin family members were downregulated in 15 dpa with expression patterns consistent with previous studies. The expansin gene plays a very important role during cell wall extension [78,79]. Ruan *et al.* found that the transcript of the fiber-specific wall loosening gene, *GhEXP1*, was high at the early phase of elongation (6 to 8 dpa), but substantially reduced to untraceable levels at 20 dpa, suggesting that at this point, the primary cell wall of elongating fiber has become very rigid, elongation has stopped because of the loss of higher turgor as a result of downregulation of transporter genes and reopening of plasmodesmata and fiber cells have shifted their energy to cellulose synthesis [79]. 

## 5. Conclusions

In this study, protocols for protein extraction from mature cotton fiber tissue were developed. Combining different protein extraction protocols and LC-MS/MS technology, we have established a comprehensive cotton fiber proteome in *Gossypium hirsutum*, including the 35 dpa fiber proteome, which has not been examined in prior reports to the best of our knowledge. Proteins with diverse functions at specific developmental stages of cotton fiber were identified by quantitative comparison at three representative developmental stages. The results provide novel insight into cotton fiber development and regulation in addition to identifying efficient methods of protein extraction for cotton fiber proteomics. 

## Supplementary Materials

The Supplementary Materials are available online at www.mdpi.com/2227-7382/4/1/7/s1.
