# Supplementary material for: Comparative Proteomic Analysis of Cotton Fiber Development and Protein Extraction Method Comparison in Late Stage Fibers
Source: Proteomes. 2016 Feb 3;4(1):7. doi: 10.3390/proteomes4010007 (PMC5217364; doi:10.3390/proteomes4010007)
Supplement: Supplementary file 1 [file proteomes-04-00007-s001.zip › proteomes-04-00007-Supplementary materials.pdf]

Supplementary Materials: Comparative Proteomic Analysis of Cotton Fiber Development and Protein Extraction Method Comparison in Late Stage Fibers

Hana Mujahid [1], Ken Pendarvis [2,†], Joseph S. Reddy [3,‡], Babi Ramesh Reddy Nallamilli [1,§], K. R. Reddy [5], Bindu Nanduri [3] and Zhaohua Peng [1,*]

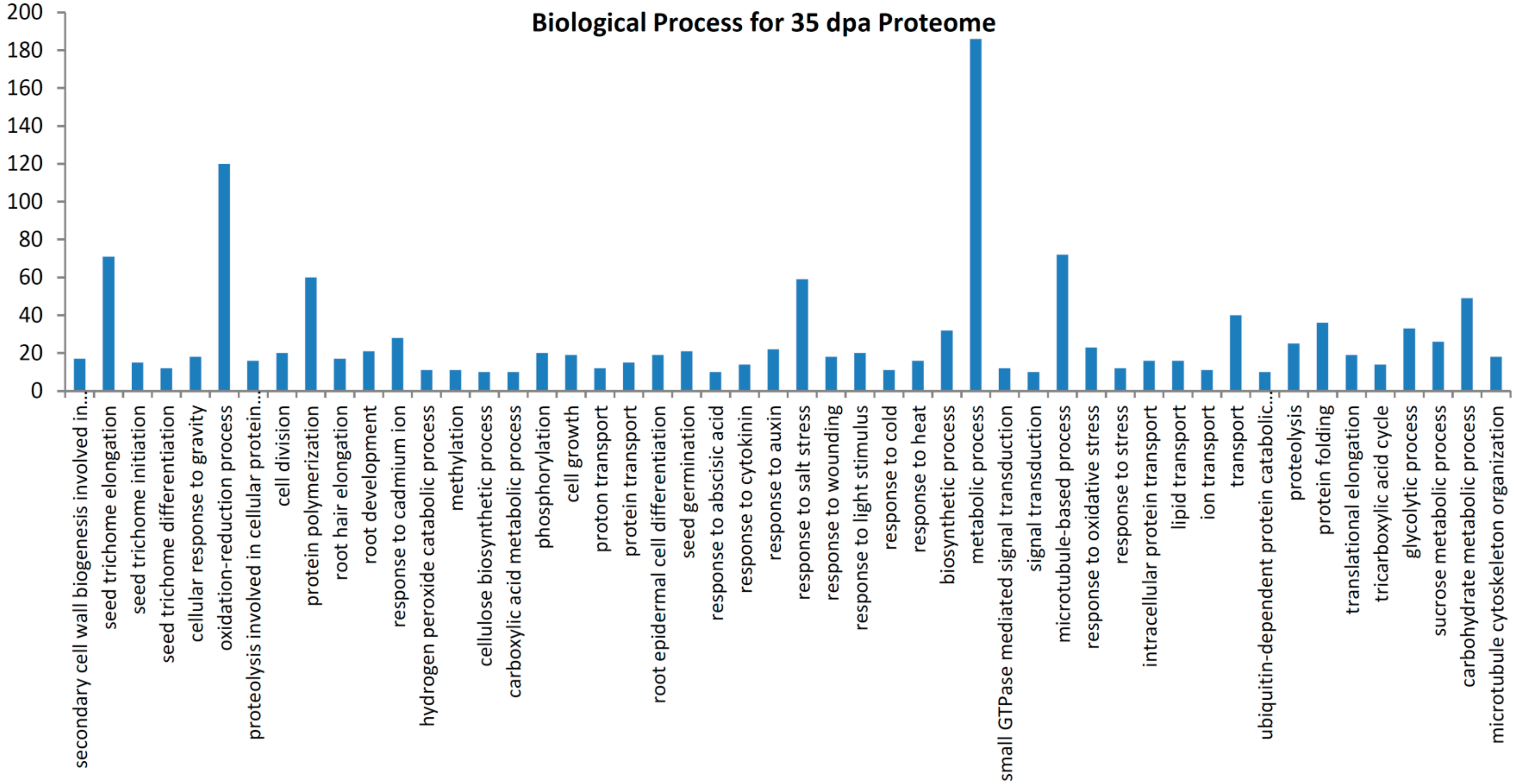

Figure S1. Predominant biological process gene ontology category terms of proteins identified in 35 dpa fiber proteome. Protein number (y axis), biological process terms (x axis). Only predominant biological process category terms are shown in figure. (…) in x axis indicates abbreviated term, complete names of abbreviated biological process GO terms from left to right include: secondary cell wall biogenesis involved in seed trichome differentiation || proteolysis involved in cellular protein catabolic process || ubiquitin-dependent protein catabolic process.

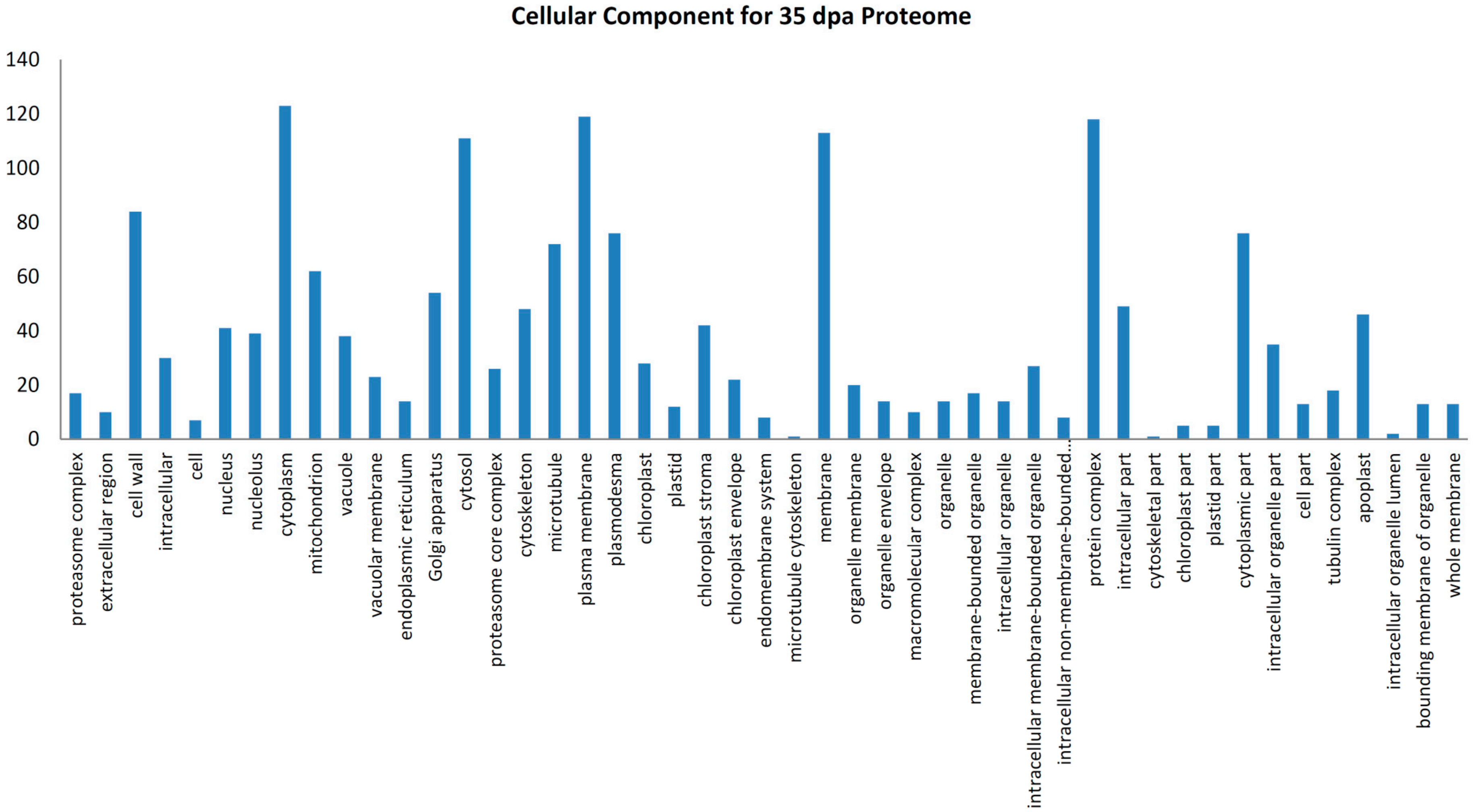

Figure S2. Predominant cellular component gene ontology category terms of proteins identified in 35 dpa fiber proteome. Protein number (y axis), cellular component terms (x axis). Only predominant cellular component category terms are shown in figure. (…) in x axis indicates abbreviated term, complete names of abbreviated cellular component GO term include: intracellular non-membrane-bounded organelle.

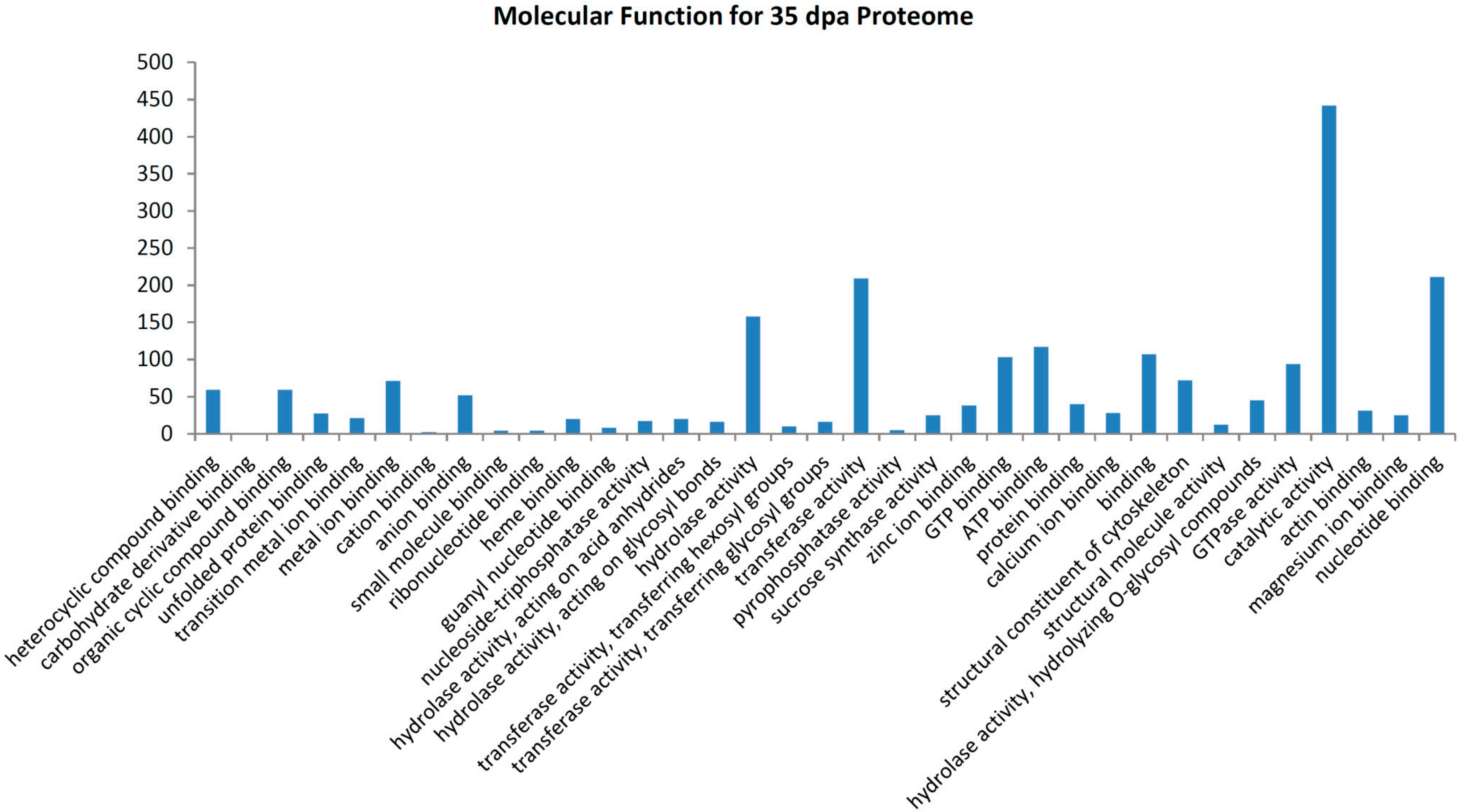

Figure S3. Predominant molecular function gene ontology category terms of proteins identified in 35 dpa fiber proteome. Protein number (y axis), molecular function terms (x axis). Only predominant molecular function category terms are shown in figure.

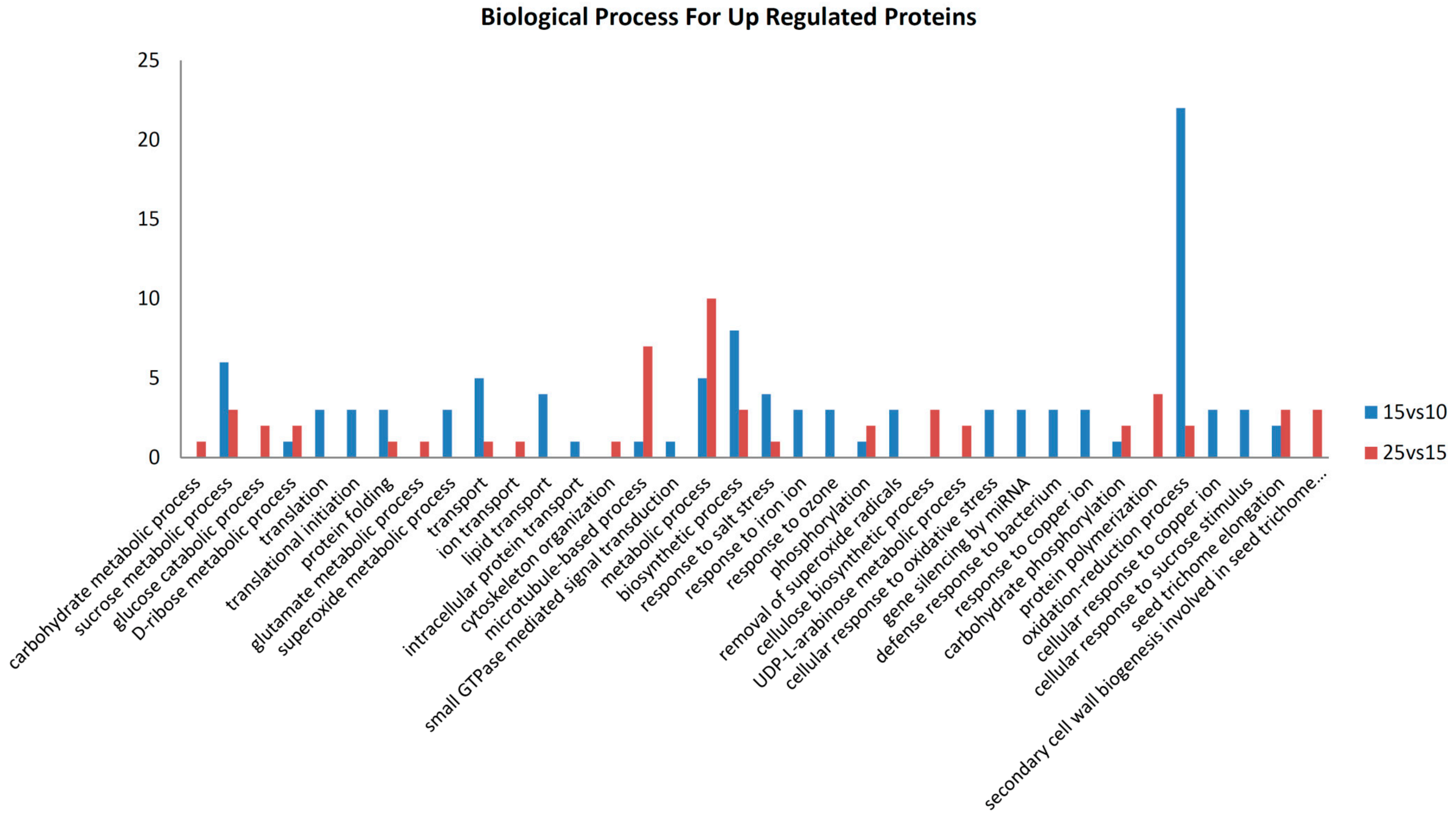

Figure S4. Predominant biological process gene ontology category terms of up regulated proteins. Protein number (y axis), biological process terms (x axis). Fiber stage comparison is indicated on the right. Only predominant biological process category terms are shown in figure. (…) in x axis indicates abbreviated term, complete name of abbreviated biological process GO term include: secondary cell wall biogenesis involved in seed trichome differentiation.

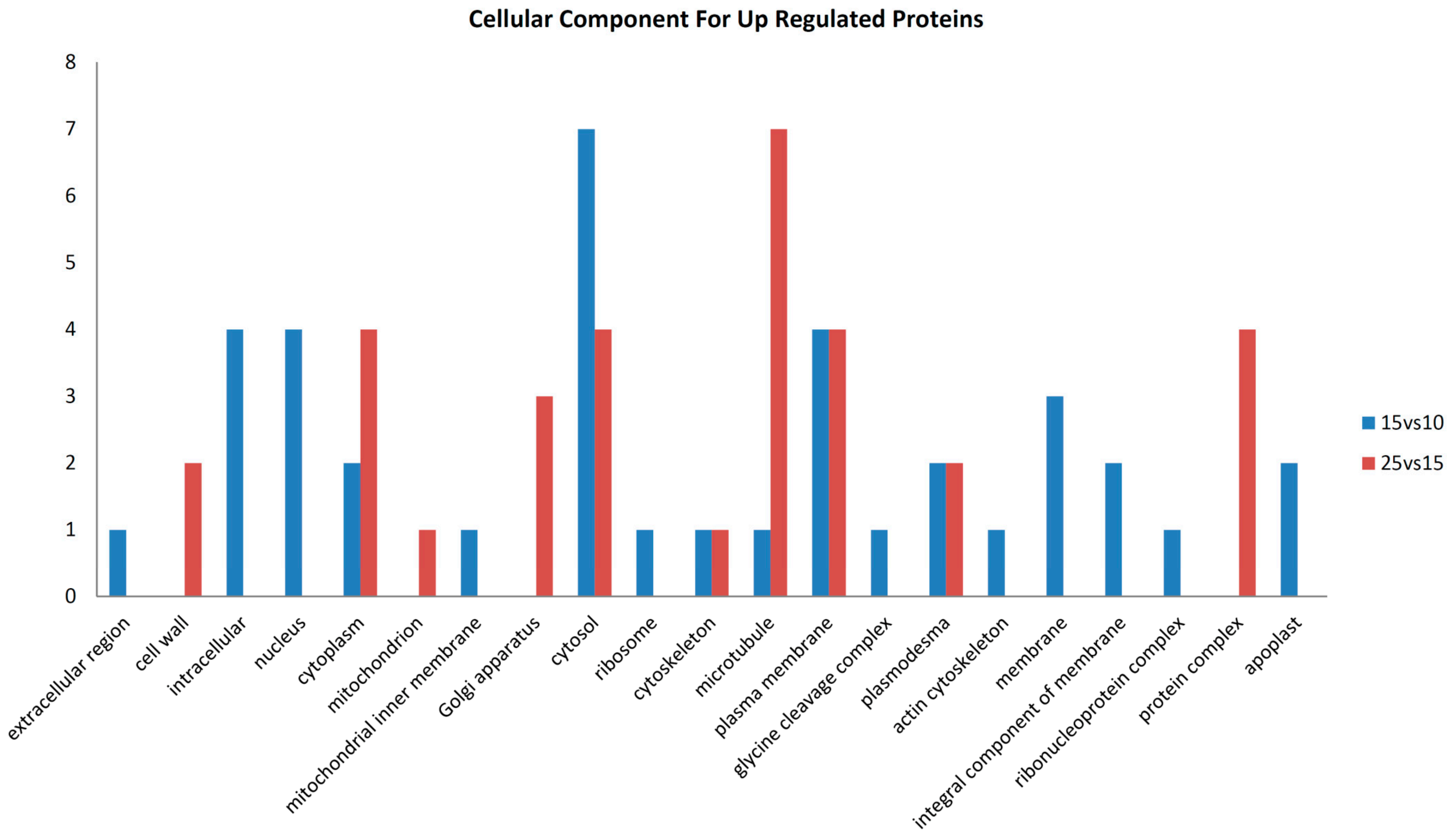

Figure S5. Predominant cellular component gene ontology category terms of up regulated proteins. Protein number (y axis), cellular component terms (x axis). Fiber stage comparison is indicated on the right. Only predominant cellular component category terms are shown in figure.

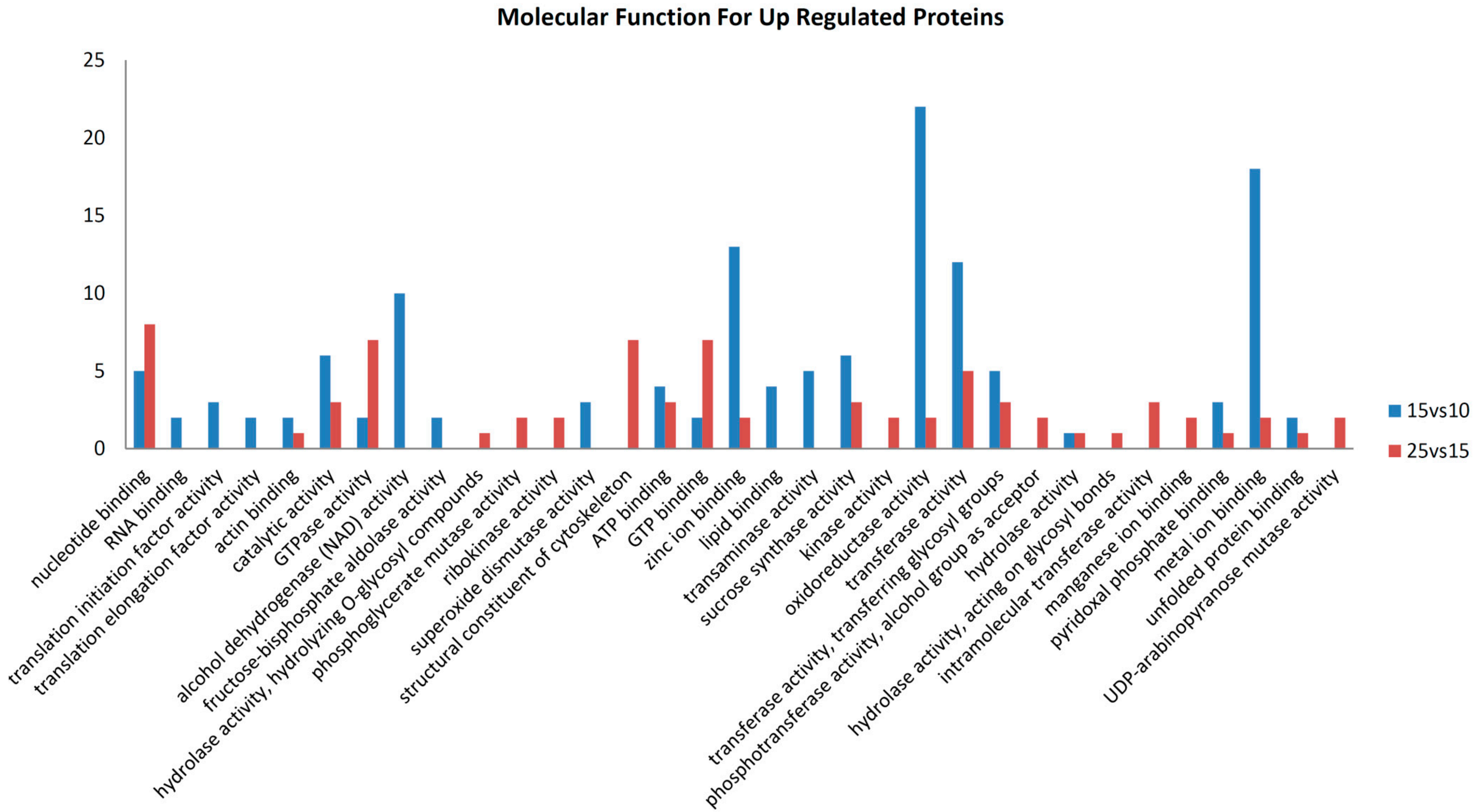

Figure S6. Predominant molecular function gene ontology category terms of up regulated proteins. Protein number (y axis), molecular function terms (x axis). Fiber stage comparison is indicated on the right. Only predominant molecular function category terms are shown in figure.

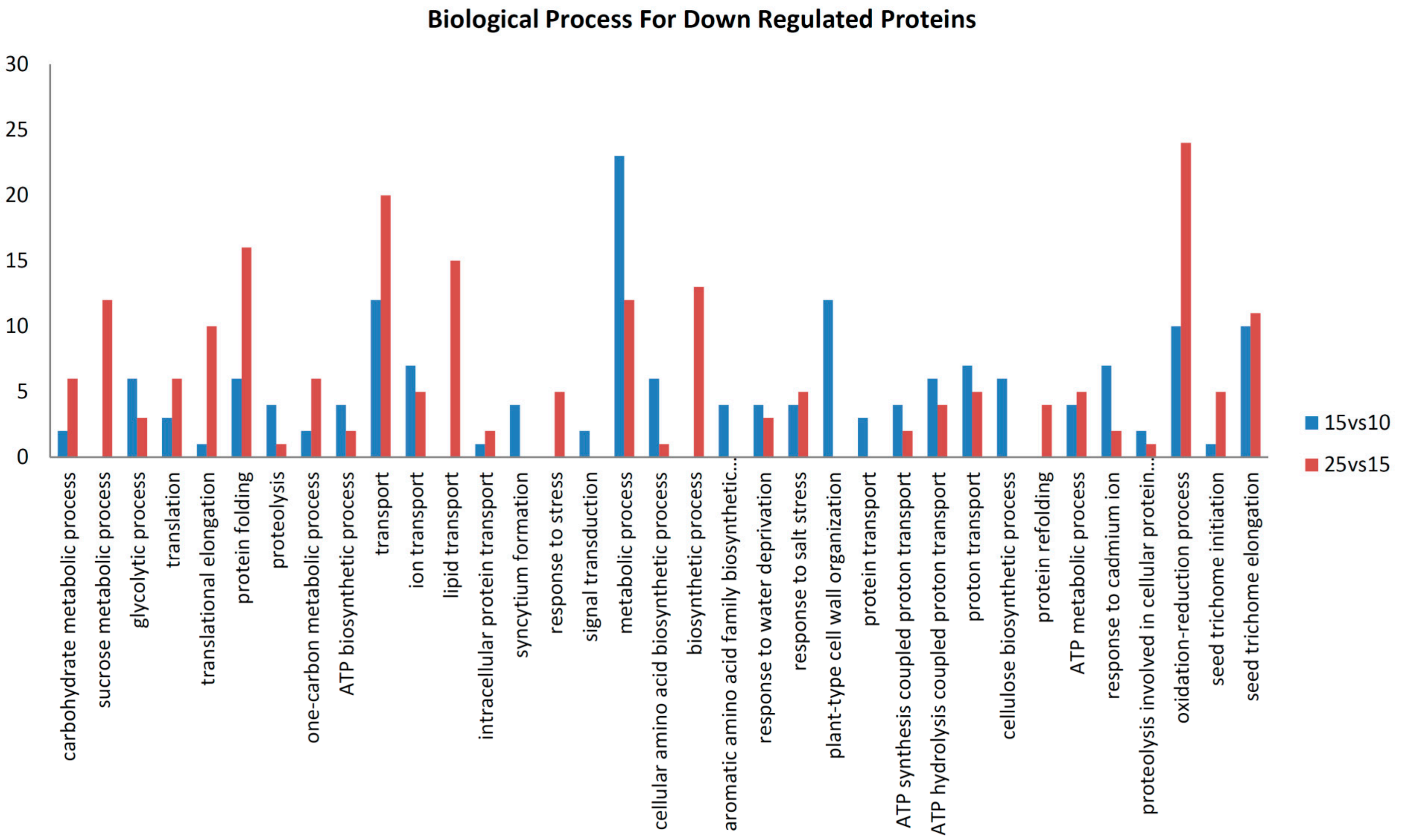

Figure S7. Predominant biological process gene ontology category terms of down regulated proteins. Protein number (y axis), biological process terms (x axis). Fiber stage comparison is indicated on the right. Only predominant biological process category terms are shown in figure. (…) in x axis indicates abbreviated term, complete names of abbreviated biological process GO terms from left to right include: aromatic amino acid family biosynthetic process || proteolysis involved in cellular protein catabolic process.

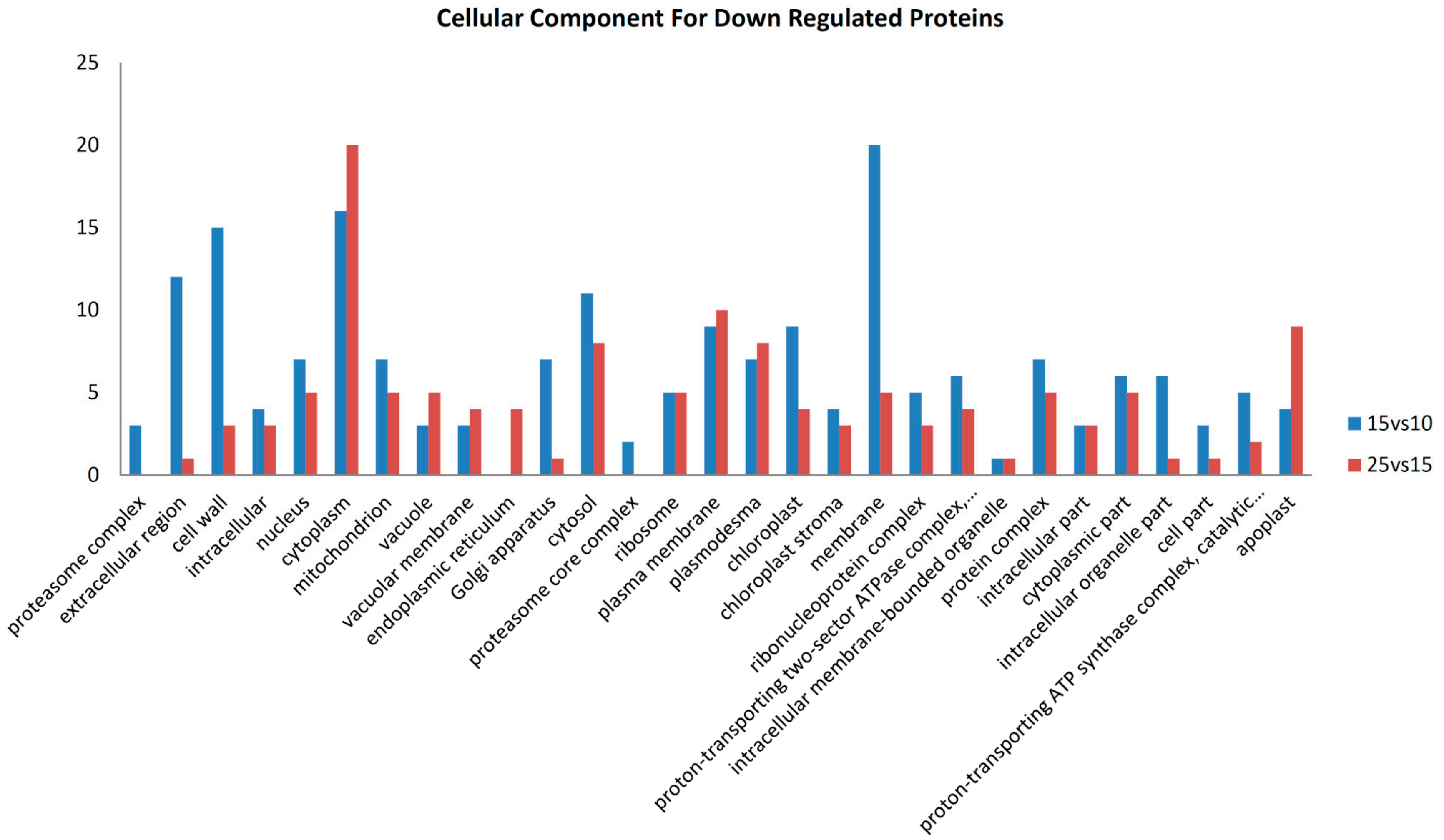

Figure S8. Predominant cellular component gene ontology category terms of down regulated proteins. Protein number (y axis), cellular component terms (x axis). Fiber stage comparison is indicated on the right. Only predominant cellular component category terms are shown in figure. (…) in x axis indicates abbreviated term, complete names of abbreviated cellular component GO terms from left to right include: proton-transporting two-sector ATPase complex, catalytic domain || proton-transporting ATP synthase complex, catalytic core F(1).

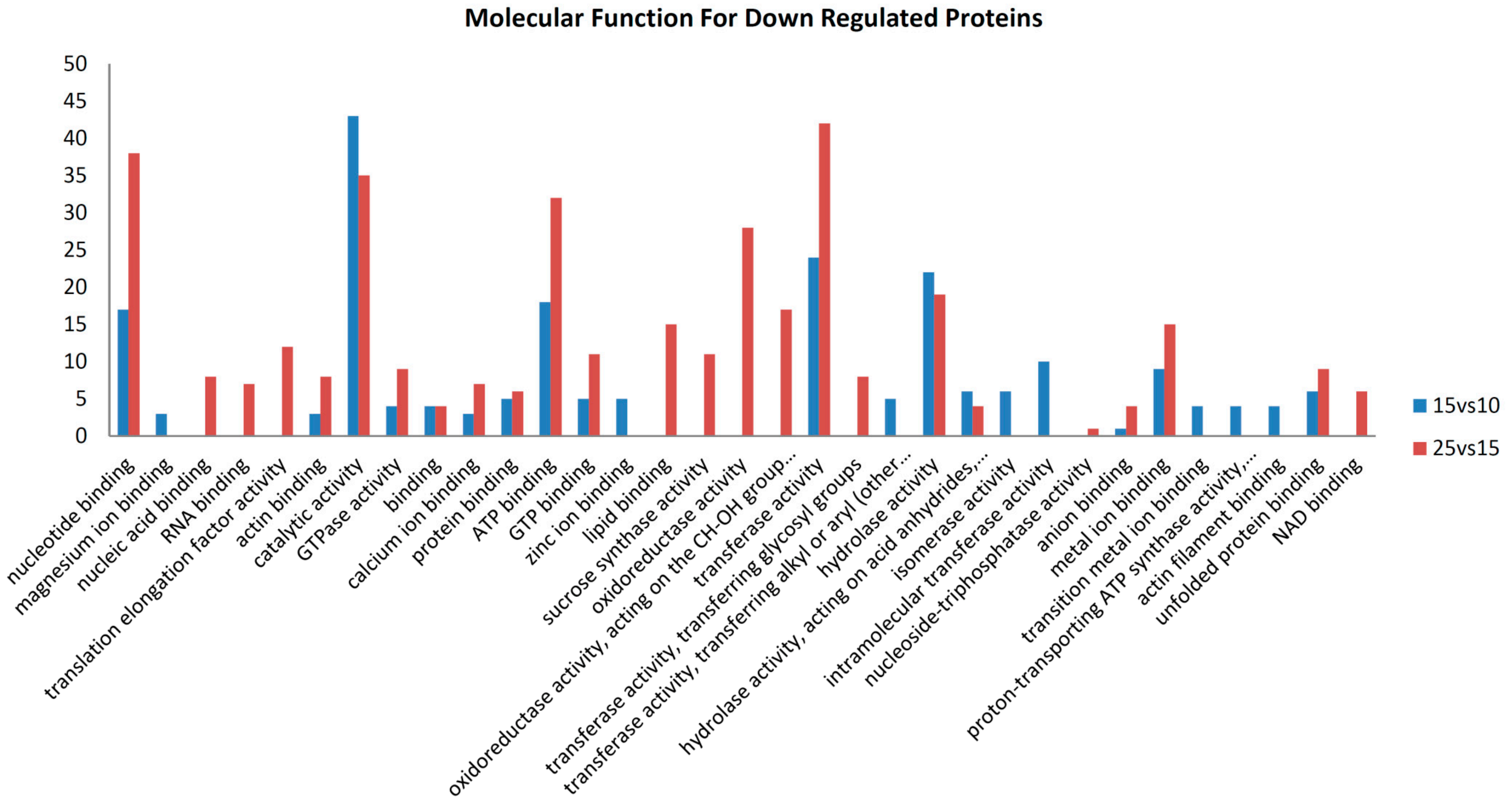

Figure S9. Predominant molecular function gene ontology category terms of down regulated proteins. Protein number (y axis), molecular function terms (x axis). Fiber stage comparison is indicated on the right. Only predominant molecular function category terms are shown in figure. (…) in x axis indicates abbreviated term, complete names of abbreviated molecular function GO terms from left to right include: oxidoreductase activity, acting on the CH-OH group of donors, NAD or NADP as acceptor || transferase activity, transferring alkyl or aryl (other than methyl) groups || hydrolase activity, acting on acid anhydrides, catalyzing transmembrane movement of substances || proton-transporting ATP synthase activity, rotational mechanism.

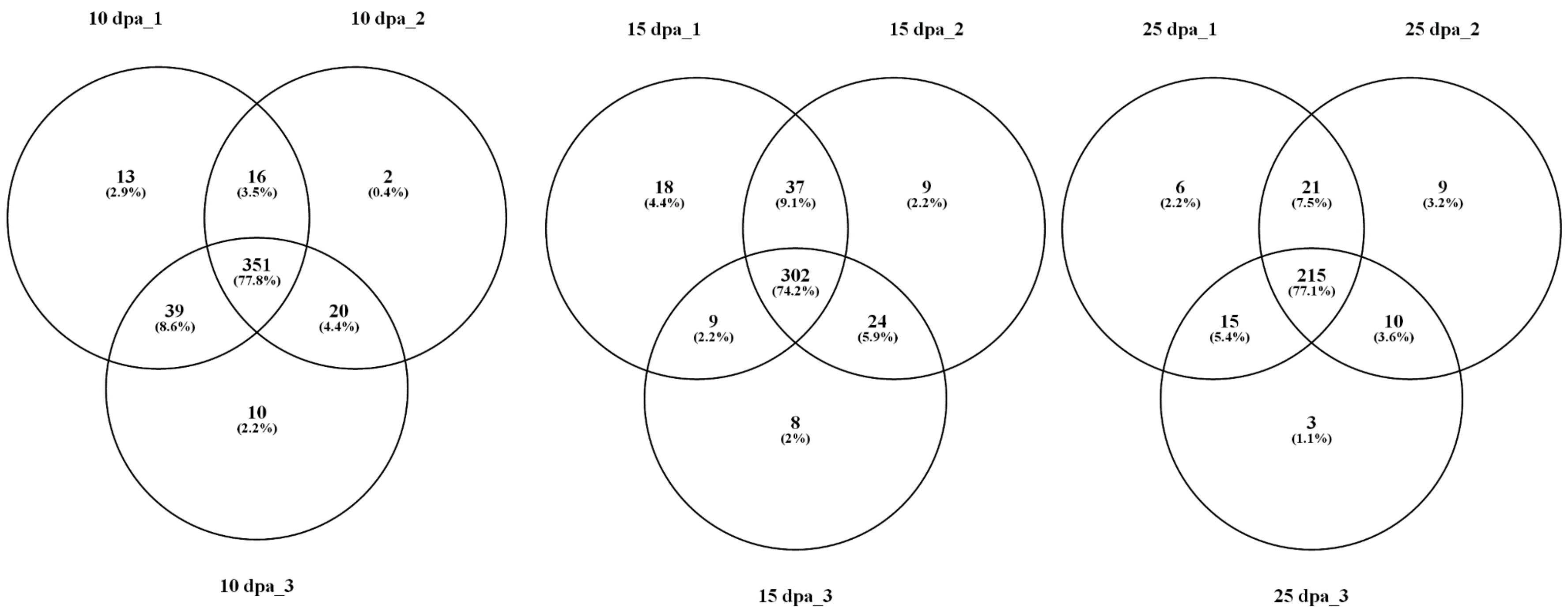

Figure S10. Venn diagrams displaying number of identified cotton proteins per replicate for 10 dpa, 15 dpa, and 25 dpa fiber extracted with phenol and the overlap of identified proteins per replicate. Venn diagrams generated using the Venny 2.1.0 tool [1].

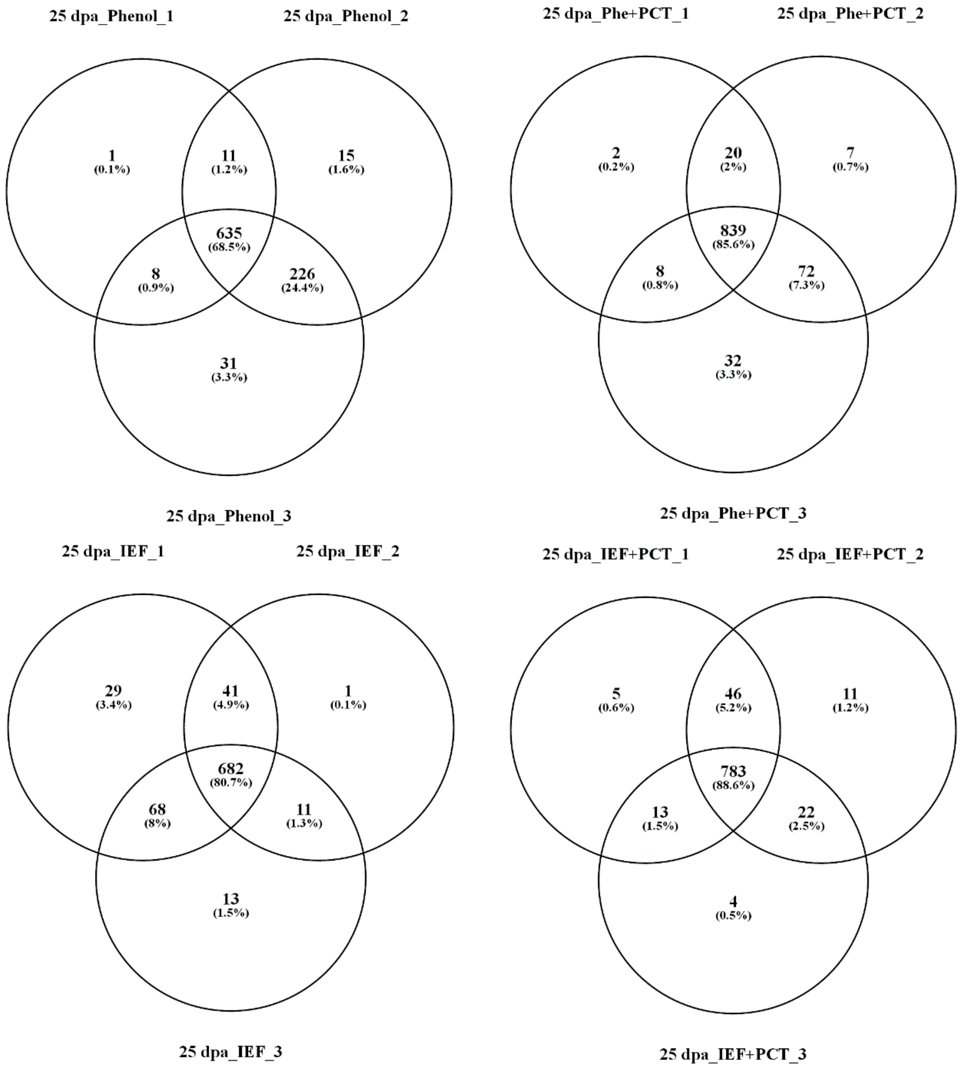

Figure S11. Venn diagrams displaying number of identified cotton proteins per replicate for 25 dpa fiber using four different extraction methods (Phenol, Phenol+PCT, IEF, and IEF+PCT) and the overlap of identified proteins per replicate. Venn diagrams generated using the Venny 2.1.0 tool [1].

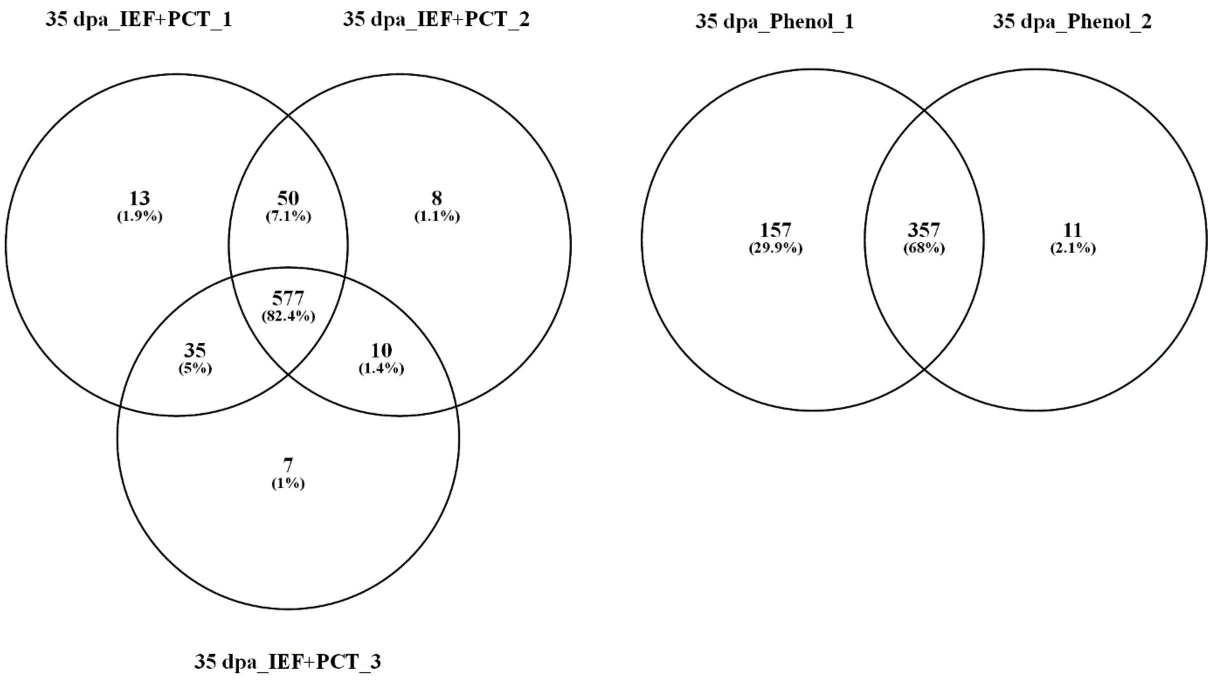

Figure S12. Venn diagrams displaying number of identified cotton proteins per replicate for 35 dpa fiber using IEF+PCT and phenol and the overlap of identified proteins per replicate. Venn diagrams generated using the Venny 2.1.0 tool [1].

Reference

1. Oliveros, J.C. (2007–2015) Venny. An interactive tool for comparing lists with Venn's diagrams. Available online: http://bioinfogp.cnb.csic.es/tools/venny/index.html (accessed on 20 May 2015).
